# Fermi level pinning characterisation on ammonium fluoride-treated surfaces of silicon by energy-filtered doping contrast in the scanning electron microscope

**DOI:** 10.1038/srep32003

**Published:** 2016-08-31

**Authors:** Augustus K. W. Chee

**Affiliations:** 1 Centre for Advanced Photonics and Electronics, Electrical Engineering Division, Department of Engineering, University of Cambridge, 9 J J Thomson Avenue, Cambridge CB3 0FA, United Kingdom

## Abstract

Two-dimensional dopant profiling using the secondary electron (SE) signal in the scanning electron microscope (SEM) is a technique gaining impulse for its ability to enable rapid and contactless low-cost diagnostics for integrated device manufacturing. The basis is doping contrast from electrical *p*-*n* junctions, which can be influenced by wet-chemical processing methods typically adopted in ULSI technology. This paper describes the results of doping contrast studies by energy-filtering in the SEM from silicon *p*-*n* junction specimens that were etched in ammonium fluoride solution. Experimental SE micro-spectroscopy and numerical simulations indicate that Fermi level pinning occurred on the surface of the treated-specimen, and that the doping contrast can be explained in terms of the ionisation energy integral for SEs, which is a function of the dopant concentration, and surface band-bending effects that prevail in the mechanism for doping contrast as patch fields from the specimen are suppressed.

The doping contrast from *p*-*n* junctions based on the secondary electron (SE) signal in the scanning electron microscope (SEM) can be optimised to support rapid and contactless two-dimensional dopant profiling, which is advantageous compared to a number of alternatives (*e.g.* secondary ion mass spectroscopy, spreading resistance profiling, atom probe tomography, scanning capacitance microscopy, *etc.*) having limited sensitivity, range or resolution, are time-consuming, costly or destructive, or provide only one-dimensional measurements. Under standard imaging conditions, the *p*-type region appears bright and the *n*-type region dark, thereby producing so-called doping contrast which is essentially a voltage contrast technique of considerable fundamental and technological importance. It is possible to use this sharp transition in brightness, which may be amplified upon an externally applied reverse bias (albeit trading off resolution), to study *p*-*n* homo- or hetero-junctions under high magnification; this has been exploited, inter alia, for process efficiency and reliability investigations on microelectronic, optoelectronic and photovoltaic devices since Oatley and Everhart (1957)^1^. Due to recent developments in SEM instrumentation, the application of doping contrast has evolved not only in physical and failure analysis of integrated circuits, critical dimension measurements on a semiconductor production line, *etc.*, but mapping of electrically active dopants at high spatial resolution and sensitivity^2^,^3^,^4^,^5^,^6^,^7^,^8^,^9^. A resolution up to 1 nm is achievable^5^,^6^ and doping contrast can be measured proportionately from dopant concentrations less than 10^14^ up to more than 10^20^ atoms cm^−3^, at a quantification accuracy of at least ± 3% 7,8.

Although an initial drawback of this technique is the lack of a complete, quantitative model, progress has been made in evaluating the doping contrast mechanism^9^, which is due to the built-in potential across the electrical junction, modified by the effects of surface band-bending, surface boundary scattering, detector collection solid angle and the local electric fields, called patch fields, above the specimen surface. Analogous to other techniques that characterise potential variations such as scanning photocurrent microscopy^10^, low-energy electro-emission^11^ or photo-emission spectroscopy^11^,^12^,^13^ *etc.*, doping contrast, being sensitive to the surface/sub-surface electric fields, can be strongly modified by any wet-chemical or electrochemical processing methods routinely employed for oxidation, etching or passivation, *etc.* during ULSI microfabrication. Doping contrast may also be produced due to ad-layer-semiconductor contacts that alter the surface band-bending depending on the doped region^14^. Hence, sufficient sensitivity and quantification accuracy cannot be reliably achieved unless the near-surface effects, including that of surface band-bending^2^,^3^,^15^ and patch fields^4^,^16^ on doping contrast are understood and well-controlled^7^,^9^. For example, to avoid surface-geometry influence from a non-homogeneous semiconductor sample that may hamper quantification via patch fields^7^,^8^, one approach may be to induce Fermi level pinning on the surface^9^, thereby resulting in surface band-bending and a surface junction potential that reduces from that in the bulk. Therefore the aim of this study is to examine and demonstrate for the first time, a uniquely innovative, rapid and facile route based on doping contrast to determine a nearly-equipotential surface of a semiconductor after wet-chemical etching, characterised by Fermi level pinning due to a high density of surface states that traps charges and removes patch fields with essentially no surface charge variation across the *p*-*n* junction.

We report herein results from silicon specimens that were surface-treated reproducibly using semiconductor-grade ammonium fluoride (NH_4_F) solution. Not only is it now commonly accepted that simple chemical etching in 40% NH_4_F is able to remove oxide layers and organic contamination, and produce atomically flat, hydrogen-terminated surfaces resulting in high quality substrates for micro- and nano-technology; it is widely believed that the resulting doping contrast change is due to an increase in patch fields owing to the hydrogen atom-passivation of dangling bonds that removes surface states and thus reduces surface band-bending^4^,^5^,^17^,^18^,^19^. However, regarding the latter we will show by specialised energy-filtering and SE micro-spectroscopy measurements in the SEM, that theoretical calculations and computer simulations actually provide a converse explanation for this doping contrast change. In those studies cited above, besides a change in the charge states, the surface state density and surface band-bending have essentially increased, leading to doping contrast commensurate with dopant concentration.

## Results

### Doping contrast changes after NH_4_F-treatment

**Figure 1:**
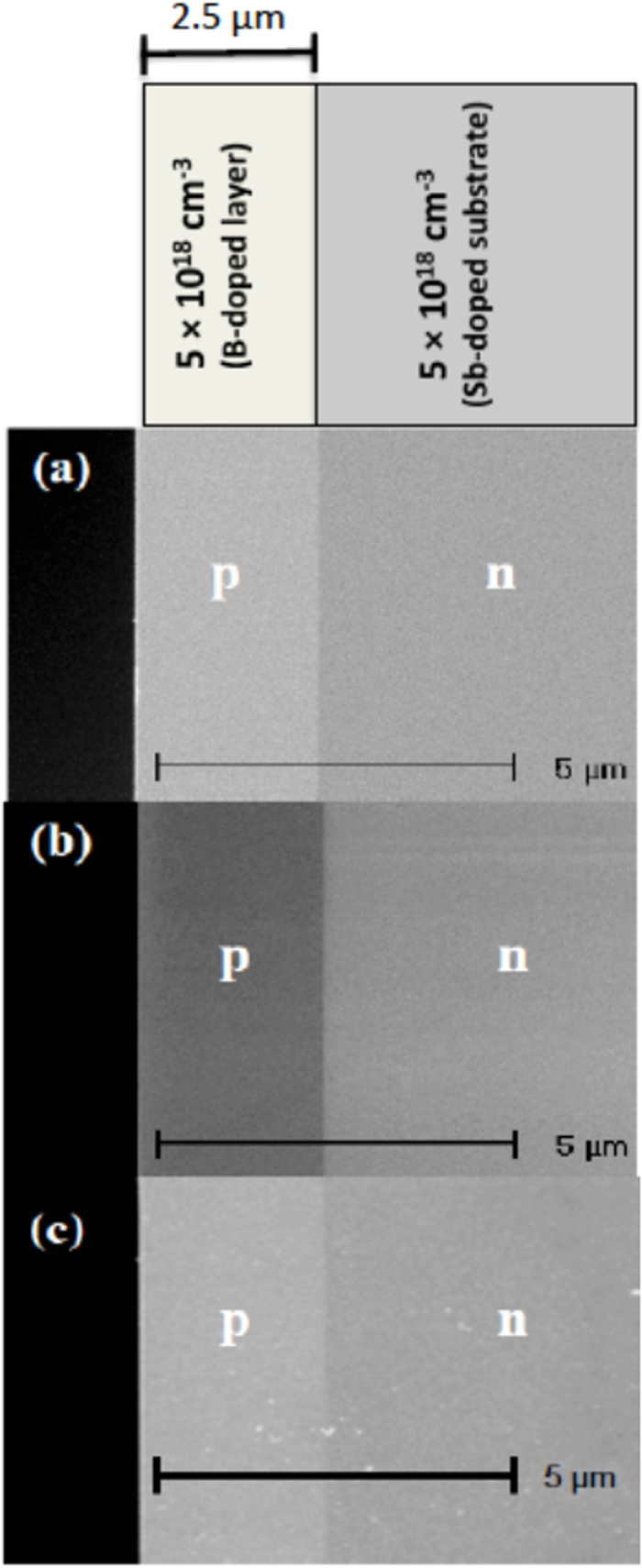
SEM doping contrast from the silicon *p*-*n* junction specimen before and after NH_4_F-treatment. Freshly-cleaved under (**a**) standard imaging conditions at *V*_*def*_ = 60 V and under (**b**) energy-filtering at *V*_*def*_ = 8 V; (**c**) after NH_4_F-treatment under standard imaging conditions (the white specks represent residual ammonium salts on the surface). Also included is a schematic showing the dopant concentrations in the specimen (measured using SIMS). The extraction potential was 250 V and working distance was 3 mm. Appropriate brightness and contrast settings were used for the respective images to demonstrate strong doping contrast.

Figure 1 draws a comparison between the doping contrast from a freshly-cleaved and NH_4_F-treated silicon *p*-*n* junction specimen. Initially, doping contrast is typical under standard imaging conditions (Fig. 1(a)) but contrast inversion (*n*-region bright and *p*-region dark) occurred under energy-filtering with a deflection voltage (*V*_*def*_) below 10 V (*e.g.* Fig. 1(b)). The freshly-cleaved specimen will have a surface layer of native oxide, about 5 to 10 Å thick, which forms rapidly after cleaving in air. Treatment with 40% NH_4_F is believed to remove this oxide layer and passivate the surface so that the oxide layer does not reform rapidly. After this surface-treatment, the specimen was immediately placed into the SEM, and the SE intensities in Fig. 1(c) were acquired within ~10 min., so the specimen almost certainly does not have an oxide layer for this image under clean, high vacuum conditions, *e.g* according to ref. 20. Line profiles across the regions of interest were row-averaged over at least 100 pixels perpendicular to the scan direction, and using the formalism detailed in ref. 9, the standard imaging doping contrast reduced from 15.7 ± 2.4% (Fig. 1(a)) to 14.3 ± 0.4% (Fig. 1(c)) after surface-treatment. The contrast magnitude is enhanced only from *p*-regions that are sufficiently highly doped (*e.g.* >10^19^ acceptors cm^−3^)^7^, or from *n*-regions so that they can be distinguished from intrinsic regions^4^. The surface states on the silicon in Fig. 1(a) compared with Fig. 1(c) are very different because of the surface-treatment, and it is this difference that must be responsible for the change in doping contrast. Energy-filtered doping contrast from NH_4_F-treated silicon *p*-*n* junction specimens has not been characterised until now. Incontrovertible differences in terms of contrast inversion or lack thereof were established under low-pass energy-filtering at low *V*_*def*_.

### SE micro-spectroscopy characterisation

**Figure 2:**
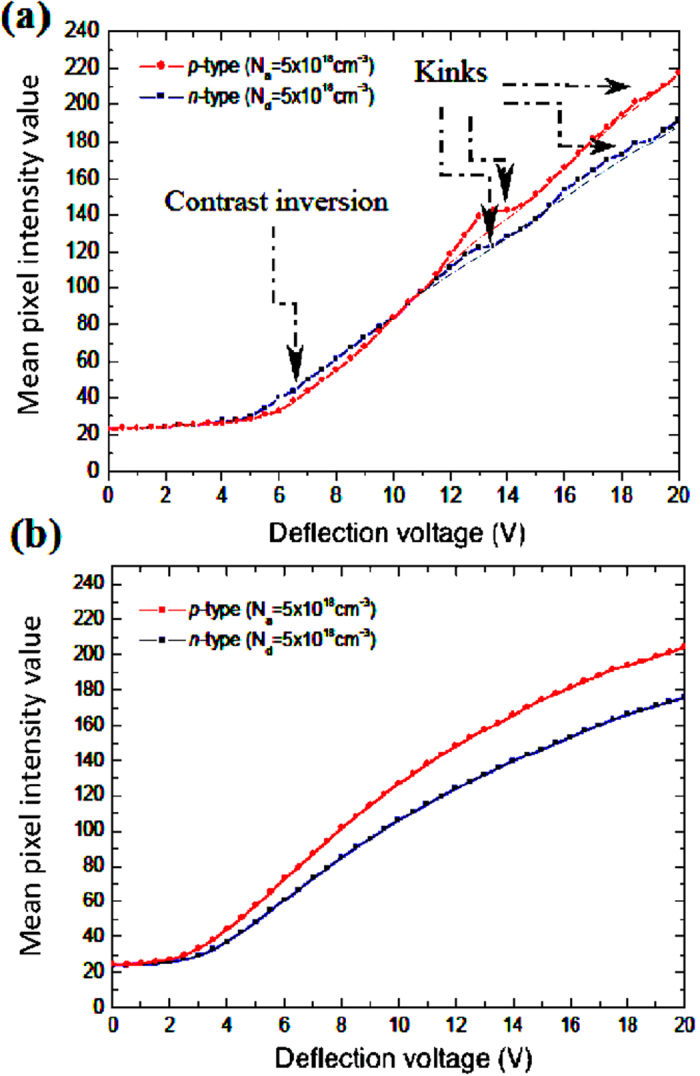
Experimental SE yield curves from the *p*- and *n*-type regions of the silicon specimen cross-section. After specimen was (**a**) freshly-cleaved, and (**b**) surface-treated. Baseline spline fits (indicated by the dash-dotted lines) are applied to highlight kinks in the yield characteristics. The error in each intensity point ranges from 2.6 to 3.4% and 0.5 to 0.6% of the intensity value for the freshly-cleaved and surface-treated sample respectively. The same brightness and contrast settings were used to allow measureable SE intensities for the entire range of deflection voltages and enable direct comparison.

**Figure 3:**
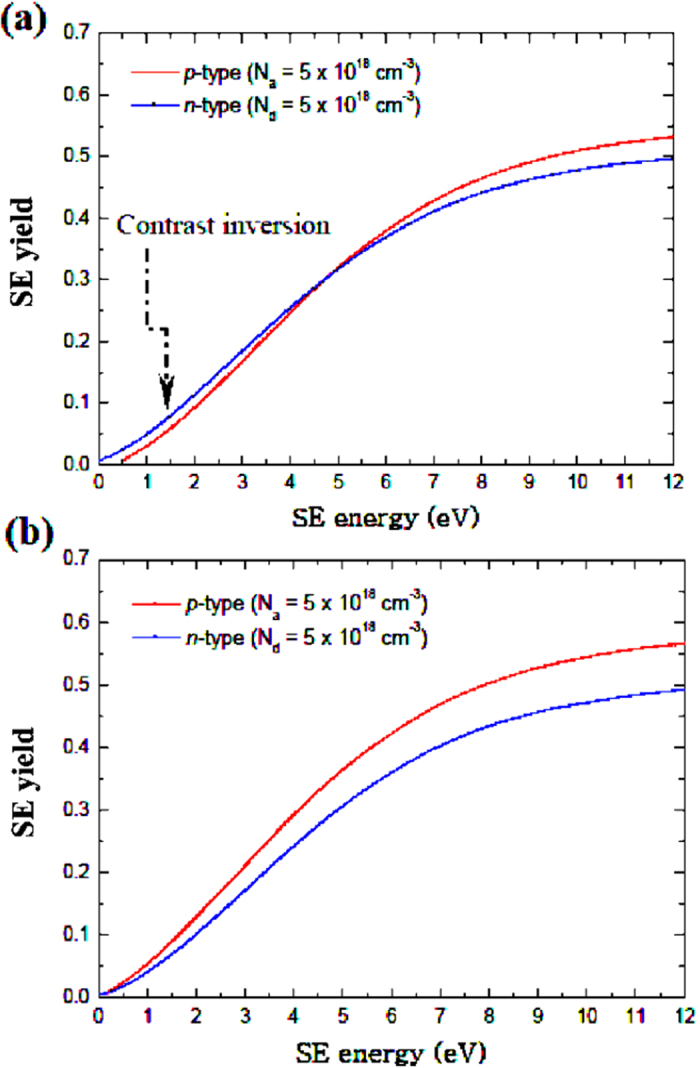
Theoretical SE yield curves from the *p*- and *n*-regions of the silicon *p*-*n* junction specimen. Calculations were for a specimen that is (**a**) freshly-cleaved and (**b**) NH_4_F-treated.

Unlike that on the as-cleaved specimen, no inversion occurred in energy-filtered doping contrast on the treated-surface, as reflected by the experimental SE yield curves (Fig. 2). The SE micro-spectroscopy acquisition was through measurements of the average pixel intensity as a function of the bias on the through-the-lens detector (TLD) deflector electrode that was swept through a specified range at fixed, but short time intervals (*e.g.* 500 ms), as documented in ref. 7 and detailed in Methods. Since contrast inversion is attributed to the presence of patch fields – which diminishes as surface bend-bending increases with the surface state density^9^ - the above result is at variance with reports in the literature of a reduced number of surface states after NH_4_F-treatment^4^,^5^,^18^,^19^. Concretely, our numerical simulations of the cumulative energy distributions of the SEs (Fig. 3) demonstrate an underpinning increase in surface state density as a factor accounting for the lack of contrast inversion. On freshly-cleaved samples with a native oxide layer, typical surface states are of the order of 10^12^ cm^−2^ 21,22,23,24 having amphoteric energy levels localised in the bandgap at 0.38 eV from the respective band edges^25^, and the electron- and hole-capture cross-sections for the surface traps are 10^−15^ and 10 cm^−2^ respectively. Where 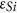 and 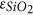 are the electric permittivities of the semiconductor and native oxide respectively, and 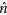 is the unit vector normal to the surface, surface band-bending was computed according to a boundary condition at the semiconductor-oxide interface as in eq. 1.


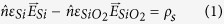


The difference between the normal components of the electric fields at the respective regions across the surface boundary must be equal to the sheet charge density 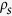
(Gauss’ Law). This charge term incorporates both the mobile and fixed charges. On silicon (110) surfaces etched in 40% NH_4_F, amphoteric surface states based on Koyanagi *et al*.^26^ have a density of 2 × 10^13^ cm^−2^, and their discrete energy levels are localised in the bandgap 0.43 eV below the conduction band edge for the donor- and acceptor-like states. This general model of monoenergetic states is consistent with the discrete-like states normally found on atomically clean surfaces^27^, as would be expected after NH_4_F-treatment of silicon.

Good agreement is observed between the experimental and numerical SE yield curves from the *p*- and *n*-regions in terms of their relative energy displacements before and after surface-treatment. The low-energy characteristics from the treated-sample signify surfaces virtually at equipotential^9^, and may confer evidence for semiconductor surface metallisation induced by atomic hydrogen^28^. Furthermore, the removal of the multiple kinks at relatively high kinetic energies after NH_4_F-treatment (see Fig. 2(b)) is striking, which *a posteriori* indicates that, far from being correctly imputed to tertiary SE3 signals originating from the pole piece or objects other than the sample as claimed in refs 29 and 30, the former are most likely associated with plasmon contributions from: oxides which form rapidly on the surface^31^; or the Si-SiO_2_ interface and/or oxide surface^32^; or beam-induced contamination on the sample. The disappearance of these effects may correspond to the dissolution of the native oxide layer or any other organic contamination, followed by passivation of the surface. The spectra in Fig. 2(a) are “kink-free” in the low kinetic energy regime (below the cross-over *V*_*def*_,) and this is *a priori* expected because the slowest SEs stem from deeper below the surface^7^,^33^. This means that by low-pass energy-filtering at an appropriate cut-off energy, it is possible to block out extraneous contributions directly from surface oxides, surface states and contamination, and thus, enhance dopant profiling quantification from as-cleaved surfaces. In fact, the treated-sample gives rise to a wider window of SE energies for superior quantification accuracy because of the removal of spurious contributions inherent in the specimen due to ambient air exposure of a previously reactive surface.

## Discussion

In our studies, we used energy-filtering and SE micro-spectroscopy measurements to elucidate the doping contrast mechanism in the SEM after etching the silicon specimens in 40% NH_4_F solution. The SE yield characterisation revealed two major features: on one hand, Fermi level pinning by a high density of extrinsic surface states on the H-terminated surface, and on the other, a contamination-free surface. Ha^12^ posited that the results of X-ray photoelectron spectroscopy support evidence for Fermi level pinning on the surface. We validated this assumption by leveraging our Monte Carlo method to compute the SE yields, which integrates a semiconductor-based finite-element model for calculating electric potential distributions inside and outside the specimen, plus a ray-tracing algorithm, as described in refs 7 and 9.

The surface-treatment is seen to have significantly increased the number of the slowest SEs emitted from the *p*-region, exceeding that from the *n*-region that is considerably reduced due to surface band-bending. The foregoing result is intuitively meaningful considering the following principle. If the surface potential energy at the *p*-region is higher (or ionisation energy is smaller), a larger proportion of the slowest SEs that arrive at the detector will originate from the *n*-region since they mostly have lower kinetic energies compared to that from the *p*-region^7^,^9^. Consequently, the yield curve from the *p*-region translates towards higher energies relative to that from the *n*-region (Figs 2(a) and 3(a)). Therefore the contrast inversion on the as-cleaved surface under appropriate energy-filtering is a corollary of this surface potential energy (or ionisation energy) difference and the attendant patch fields that influence the SE angular distributions. Further elaboration of this result is given in ref. 9. The observation that no contrast inversion occurs after surface-treatment strongly pleads for the case of a dramatically reduced ionisation energy difference at the surface. Had the surface state density been reduced, it would, in turn, have reduced the surface band-bending. This in turn would have increased the difference in ionisation potential energies at the surface between the two sides of the *p*-*n* junction according to *e.g.* ref. 4, thus resulting in contrast inversion upon passing through only the slowest SEs.

Our experimental results here and others’^13^,^26^,^34^ are in concordance with our expectations that for the surface-treatment procedure used here, the silicon (110) surface had been etched, and any ammonium salt deposits on the hydrogen-terminated surface may contribute to an increase in the extrinsic surface state density due to the adsorbent-induced states. Ongoing work is currently focussed on experimentally calibrating the simulation model to reliably provide quantitative determination of the exact shapes and sizes of the SE spectra under all operating conditions of the SEM, including the low energy offset and the kinks in the relatively high energy range. Besides correcting for the solid collection angle, this may also necessitate taking into consideration a non-ideal modulation transfer function (MTF) and detective quantum efficiency (DQE) of the TLD scintillator. With the origin of kinetic energy estimated to be centred on *V*_*def*_  ≈ 1.5 V, there is clearly no contrast over a small range of deflector biases up to about 5 V for the freshly-cleaved sample. This may be because of attenuation of the secondary emission signal (that stems from deeper below the surface) by any surface oxides that the Monte Carlo simulations do not account for since no appropriate scattering models for ultra-thin native oxide coverages are available at present. Nevertheless, the effect of the native oxide layer on the potential is essentially irrelevant in the calculations since the electron probe in the SEM during operation completely penetrates the thin (~0.2 to 1 nm) surface oxide layer of the freshly-cleaved silicon, modulating its conductivity such that the surface of the over-layer assumes the same potential as that of the semiconductor surface^35^. Any surface contamination or micro-roughness is also not modelled, and therefore as expected, the theoretical SE spectra exhibit no irregular kinks that are inimitable characteristics of the experimental yield curves from the as-cleaved specimen at the relatively high kinetic energies.

In summary, the application of a novel, highly sensitive and straightforward SEM doping contrast technique to directly determine Fermi level pinning on the surface was demonstrated. Whilst appropriate treatment with NH_4_F facilitates the preparation of contamination-free, hydrogen atom-passivated surfaces that may be desirable for device fabrication, this also benefits dopant profiling quantification accuracy in the SEM. Through specialised energy-filtering techniques and numerical simulations, we showed that doping contrast from silicon samples that were etched in 40% NH_4_F is primarily governed by surface band-bending effects whilst patch fields are diminished; a near equipotential surface is created after surface-treatment, with an increased density of trapped surface charges and Fermi level pinning. These findings also show that the enhanced doping contrast reported by Sealy *et al*.^4^, Elliot *et al*.^5^,^18^, Müllerová *et al*.^17^ and Lin and Lee^19^, is not due to a reduction in surface state density and surface bend-bending, but rather, to the contrary.

## Methods

### Doping contrast from *p*-*n* junction specimens

The monocrystalline silicon specimens in this study comprise a symmetric *p*-*n* junction with a 2.5 μm thick *p*-layer, chemical vapour deposition (CVD)-grown with dopant incorporation at 5 × 10^18^ B atoms cm^−3^ onto an Sb-doped *n*-substrate (Fig. 1). This was along the [001] direction at a growth rate of ~0.15 μm/min., at an operating temperature of ~1123 K and atmospheric pressure. SE doping contrast and micro-spectroscopy measurements were performed a) on silicon (110) cross-sections of the specimens after they were freshly-cleaved in air, and b) directly after NH_4_F etching of the specimens in a), when inserted into the vacuum chamber at a base pressure of ~3 × 10^−6^ mbar using an oil-free turbo-pump system; at least five repeated measurements were made on each sample. Great care was taken to ensure that the cleaved specimen exhibited a mirror-like reflective surface with no evident steps in the regions of interest. The samples were dipped into freshly-prepared 40% NH_4_F (ARISTAR™) for ~1 min, using PTFE tweezers, before they were rinsed thoroughly in semiconductor-grade deionised (DI) water for ~3 –4 min. The highly hydrophobic nature implies that the silicon surface was at least partially hydrogen-terminated.

The specimen stage was aligned so that the electron beam was incident normally on the semiconductor cross-section and the doping junction was orthogonal to the direction of the raster scan. An optimised set of beam parameters was used with an objective aperture of 30 μm diameter: a 1 kV primary electron probe having a current of ~32 pA and a probe diameter of ~16 nm. These probe conditions are known to maximise doping contrast, at least from freshly-cleaved cross-sections^7^,^8^. All the images (712 × 484 pixels) were digitally acquired at a magnification of 6,500× and a scan frequency of ~0.1 frame s^−1^ (the doped regions of interest were scanned only once), and stored as 8-bit datasets. The doping contrast data were processed using a Java plug-in written for ImageJ^TM^^36^.

The SEM was an FEI XL30^TM^ Schottky field emission gun (sFEG) equipped with an SE detection system that combines a TLD with an energy filter that is available in many modern instruments. The extraction potential was 250 V and working distance was 3 mm. Under standard ultra-high resolution (UHR) imaging conditions, image formation and doping contrast are derived from SEs of all energies (up to 50 eV), which are trapped on-axis by the strong objective lens magnetic fields and pass up through the lens bore at a rate depending on the extraction field. Under low-pass energy-filtering, *V*_*def*_ sets the maximum kinetic energy for the SEs that are allowed to pass through to the scintillator to be collected and contribute to the image.

### SE micro-spectroscopy characterisation

Further elaboration of energy-filtering with the TLD can be found in ref. 29, which can be employed for micro-spectroscopy measurements by means of varying *V*_*def*_ in definite steps at judiciously chosen, regular time intervals. Fine energy resolution is enabled by a custom-built DC power supply that can configure discrete steps as small as ±30 mV to bias the deflector electrode over a dynamic range from 0 to 20 V^7^,^29^. Automated control of *V*_*def*_ was through the National Instruments (NI) LabVIEW 7^TM^ software, and a frame grabber card was used to feedback the generated image onto a separate display screen for measurements. For cumulative SE spectra measurements, the intensity value at each bias step is the energy integral of the yield spectrum up to the maximum kinetic energy limit set by *V*_*def*_. The data were processed from box-averaging over at least 40 × 40 pixels within the scanned field areas of interest at a TV scan frequency of 1 frame_8 × _s^−1^ (frame_8 × _refers to 8 averaged frames), and recorded in real-time against *V*_*def*_ at fixed time intervals. Whilst *V*_*def*_ was ramped from 20 to 0 V in −0.5 V steps, beam-induced extraneous effects were kept minimal as data acquisition was performed rapidly, within a total timescale of 20 s; essentially the same spectra were obtained when sweeping *V*_*def*_ in the reverse direction from 0 to 20 V, indicating that temporal control of the voltage steps was optimised to avoid statistical fluctuations in the measurements due to contamination and/or charging by the electron probe interaction. A delicate balance between accuracy and throughput is struck with a sweep rate of 2 Hz as a sensible maximum, limited by the signal-to-noise ratio, and system response time taking into account, *e.g.* the slew rate and settling time of the data converters and electronic amplifiers involved.

### Numerical simulations

Solutions to the semiconductor Poisson (Laplace) equation for a semiconductor-(oxide-) vacuum system were self-consistently obtained by the finite-element method through an iterative procedure that satisfies a convergence criterion, thereby providing the equilibrium carrier and electrostatic field distributions. Surface band-bending was computed from the modified boundary condition between the semiconductor and the hydrogenated surface layer. In the foregoing calculations, the spatial distribution of the surface states before or after surface-treatment was assumed to be uniform in the semiconductor plane, and independent of doping. When computing the ratio and angular distribution of the SEs, a quantum-mechanical model for transmission, reflection or refraction was employed to describe electron scattering at the surface boundary of the solid. A generic, geometric finite-element model integrating the TLD (provided in private communication with FEI company) was incorporated in these simulations, including realistic descriptions of the immersion lens electromagnetic and electrostatic fields in the electron optical column and the specimen chamber of the SEM; hence *e.g.*, embodying the working distance- and bias-dependent variables, *etc.* Extraction and deflection field conditions equivalent to that adopted in the experiments were accounted for and specified, and 100% internal detection efficiency was assumed for the TLD.

## Additional Information

**How to cite this article**: Chee, A. K. W. Fermi level pinning characterisation on ammonium fluoride-treated surfaces of silicon by energy-filtered doping contrast in the scanning electron microscope. *Sci. Rep.* **6**, 32003; doi: 10.1038/srep32003 (2016).

## References

[b1] Oatley C. W. & Everhart T. E. The examination of p-n junctions with the scanning electron microscope. Journal of Electronics 2, 568–570 (1957).

[b2] Perovic D. D. . Field-emission SEM imaging of compositional and doping layer semiconductor superlattices. Ultramicroscopy 58, 104–113 (1995).

[b3] Perovic D. D., Turan R. & Castell M. R. Quantitative imaging of semiconductor doping distributions using a scanning electron microscope. In “The Electron”, Proceedings of the International Centennial Symposium on the Electron, Cambridge, United Kingdom, 1997, *Book 687, edited by* Kirkland A. & Brown P. D., 258–265 (IOM Communications Ltd, London, 1998).

[b4] Sealy C. P., Castell M. R. & Wilshaw P. R. Mechanism for secondary electron dopant contrast in the SEM. Journal of Electron Microscopy 49, 311–321 (2000). 11108054 10.1093/oxfordjournals.jmicro.a023811

[b5] Elliot S. L., Broom R. F. & Humphreys C. J. Dopant profiling with the scanning electron microscope – A study of Si. Journal of Applied Physics 91, 9116 (2002).

[b6] Chee A. K. W., Rodenburg C. & Humphreys C. J. High resolution dopant profiling in the SEM, image widths and surface band-bending. In Journal of Physics: Conference Series 126, 012033 (IOP Publishing, 2008).

[b7] Chee A. K. W. *Novel investigations of contrast in the scanning electron microscope towards a new generation of* doping profiling techniques engineered for semiconductor (opto)electronic device technology. PhD thesis (University of Cambridge, Cambridge, United Kingdom, 2009).

[b8] Chee A. K. W. Quantitative dopant profiling by energy-filtering in the scanning electron microscope. IEEE Transactions on Device and Materials Reliability 16, 138–148 (2016).

[b9] Chee A. K. W., Broom R. F., Humphreys C. J. & Bosch E. G. T. A quantitative model for doping contrast in the scanning electron microscope using calculated potential distributions and Monte Carlo simulations. Journal of Applied Physics 109, 013109 (2011).

[b10] Ahn Y. H., Tsen A. W., Kim B., Park Y. W. & Park J. Photocurrent imaging of p−n junctions in ambipolar carbon nanotube transistors. Nano Letters 7, 3320–3323 (2007). 17939725 10.1021/nl071536m

[b11] Piccardo M. . Low-energy electro- and photo-emission spectroscopy of GaN materials and devices. Journal of Applied Physics 117, 112814 (2015).

[b12] Ha S. *Electrical properties of surface-modified silicon nanomembranes*. PhD Thesis (University of Wisconsin- Madison, Wisconsin, United States (2008).

[b13] Schlaf R., Hinogami R., Fujitani M., Yae S. & Nakato Y. Fermi level pinning on HF etched silicon surfaces investigated by photoelectron spectroscopy. Journal of Vacuum Science & Technology A: Vacuum, Surfaces, and Films 17, 164 (1999).

[b14] El-Gomati M. M., Wells T. C. R., Müllerová I., Frank L. & Jayakody H. Why is it that differently doped regions in semiconductors are visible in low voltage SEM? IEEE Transactions on Electron Devices 51, 288–292 (2004).

[b15] Castell M. R. . (1995). Topographical, compositional, and dopant contrast from cleavage surfaces of GaAs AlxGa1xAs superlattices. In Journal of Physics: Conference Series 146, 281–284 (IOP Publishing, 1995).

[b16] Howie A. Recent developments in secondary electron imaging. Journal of Microscopy 180, 192–203 (1995).

[b17] Müllerová I., El-Gomati M. M. & Frank L. Imaging of the boron doping in silicon using low energy SEM. Ultramicroscopy 93, 223–243 (2002). 12492233 10.1016/s0304-3991(02)00279-6

[b18] Elliott S. L. *Dopant profiling with the scanning electron microscope.* PhD thesis (University of Cambridge, Cambridge, United Kingdom, 2001).

[b19] Lin C. Y. & Lee J. H. Enhanced SEM doping contrast. In Proceedings of the 29th International Symposium for Testing and Failure Analysis, Santa Clara, California, USA, 87–89 (ASM International, Ohio, 2003).

[b20] Gonda S., Tanaka M., Kurosawa T. & Kojima I. Sub-nanometer scale measurements of silicon oxide thickness by spectroscopic ellipsometry. Japanese Journal of Applied Physics 37, L1418–L1420 (1998).

[b21] Yamagishi H. Fermi level stabilization and surface states at the interfaces of Si(111) surfaces and insulating layers. Journal of the Physical Society of Japan 25, 766–773 (1968).

[b22] Poindexter E. H. . Electronic traps and Pb centers at the Si/SiO2 interface: Band-gap energy distribution. Journal of Applied Physics 56, 2844 (1984)

[b23] Brower K. L. & Headley T. J. Dipolar interactions between dangling bonds at the (111) Si-SiO_2_ interface. Physical Review B 34, 3610–3619 (1986). 10.1103/physrevb.34.3610 9940122

[b24] Cartier E. & Stathis J. H. Hot-electron induced passivation of silicon dangling bonds at the Si(111)/SiO_2_ interface. Applied Physics Letters 69, 103 (1996).

[b25] Oskam G., Hoffmann P. M., Schmidt J. C. & Searson P. C. Energetics and kinetics of surface states at n-type silicon surfaces in aqueous fluoride solutions. The Journal of Physical Chemistry 100, 1801–1806 (1996).

[b26] Koyanagi S., Hashizme T. & Hasegawa H. Contactless characterization of thermally oxidized, air-exposed and hydrogen-terminated silicon surfaces by capacitance-voltage and photoluminescence methods. Japanese Journal of Applied Physics 35, 946–953 (1996).

[b27] Allen F. G. & Gobeli G. W. Work function, photoelectric threshold, and surface states of atomically clean silicon. Physical Review 127, 150–158 (1962).

[b28] Derycke V. . Nanochemistry at the atomic scale revealed in hydrogen-induced semiconductor surface metallization. Nature Materials 2, 253–258 (2003). 12690399 10.1038/nmat835

[b29] Kazemian P., Mentink S. A. M., Rodenburg C. & Humphreys C. J. High resolution quantitative two-dimensional dopant mapping using energy-filtered secondary electron imaging. Journal of Applied Physics 100, 054901 (2006).

[b30] Kazemian P., Mentink S. A. M., Rodenburg C. & Humphreys C. J. Quantitative secondary electron energy filtering in a scanning electron microscope and its applications. Ultramicroscopy 107, 140–150 (2007). 16872746 10.1016/j.ultramic.2006.06.003

[b31] Reimer L. Scanning electron microscopy: Physics of image formation and microanalysis. (Springer-Verlag Berlin and Heidelberg GmbH & Co. K, 1998).

[b32] Komoda H. . Selective excitation of a symmetric interference plasmon mode in two close planar SiO_2_/Si interfaces observed by electron energy-loss spectroscopy. Japanese Journal of Applied Physics 40, 4512–4515 (2001).

[b33] Koshikawa T. & Shimizu R. A Monte Carlo calculation of low-energy secondary electron emission from metals. Journal of Physics D: Applied Physics 7, 1303–1315 (1974).

[b34] Angermann H., Rappich J., Sieber I., Hübener K. & Hauschild J. Smoothing and passivation of special Si(111) substrates: Studied by SPV, PL, AFM and SEM measurements. Analytical and Bioanalytical Chemistry 390, 1463–1470 (2007). 18066540 10.1007/s00216-007-1738-5

[b35] Everhart T. E., Wells O. C. & Matta R. K. Evaluation of passivated integrated circuits using the scanning electron microscope. Journal of the Electrochemical Society 111, 929 (1964).

[b36] Schneider C. A., Rasband W. S. & Eliceiri K. W. NIH Image to ImageJ: 25 years of image analysis. Nature Methods 9, 671–675 (2012). 22930834 10.1038/nmeth.2089 PMC5554542

